# Supplementary material for: Predicting the Impact of Polysulfone Dialyzers and Binder Dialysate Flow Rate on Bilirubin Removal
Source: Bioengineering (Basel). 2024 Dec 12;11(12):1262. doi: 10.3390/bioengineering11121262 (PMC11673171; doi:10.3390/bioengineering11121262)
Supplement: Supplementary file 1 [file bioengineering-11-01262-s001.zip › bioengineering-3307980-supplementary.pdf]

Supplementary Materials

Table S1. Toxin concentrations in blood analog solution.

Solute	Concentration
BSA	2 g/dL [18]
Bilirubin	20 mg/dL [70, 71]
Cholic Acid	19.3 μM [72]
Creatinine	15 mg/dL [73]
Indoxyl Sulfate	4 mg/dL [74]
Copper	21.56 μM [75]
Manganese	2.5 μM [75]

Table S2. Hydraulic Permeability Measurements from Individual Trials. *Lp* refers to hydraulic permeability. $P_{b,in}$ refers to blood side (luminal) inlet pressure. $P_{b,out}$ refers to blood side outlet pressure. ΔV refers to the change in volume. The test highlighted in bold was an outlier which was discarded.

Lp (m/s×Pa)	Mini-Module	Flow Rate (μL/min)	$P_{b,in}$ (cm H2O)	$P_{b,out}$ (cm H2O)	ΔV (mL)	Time (min)
1.013×10^{-10}	F6HPS2	1910	209.7	3.94	0.628	10.5
1.020×10^{-10}	F6HPS2	764	65.139	2.615	0.383	20
9.601×10^{-11}	F6HPS2	382	35.828	2.58	0.409	40
2.768×10^{-11}	F6HPS2	191	19.76	4.162	0.11	60
8.851×10^{-11}	F6HPS2	191	21.127	2.904	0.473	80
9.750×10^{-11}	F6HPS	1910	531.57	3.726	1.45	10
7.463×10^{-11}	F6HPS	764	269.35	1.955	1.122	20
4.257×10^{-11}	F6HPS	191	50.8	4.507	0.522	80

Table S3. Average and standard deviation hydraulic permeability values for both mini-modules and overall.

Mini-Module	Average *Lp* (m/s×Pa)	Standard Deviation (m/s×Pa)
F6HPS	7.157×10^{-11}	2.75928×10^{-11}
F6HPS2	9.696×10^{-11}	6.233×10^{-12}
Total	8.61×10^{-11}	2.14×10^{-11}
